# Research capacity, motivators and barriers to conducting research among healthcare providers in Tanzania’s public health system: a mixed methods study

**DOI:** 10.1186/s12960-023-00858-w

**Published:** 2023-09-05

**Authors:** James T. Kengia, Albino Kalolo, David Barash, Cindy Chwa, Tuna Cem Hayirli, Ntuli A. Kapologwe, Ally Kinyaga, John G. Meara, Steven J. Staffa, Noor Zanial, Shehnaz Alidina

**Affiliations:** 1 Department of Health, Nutrition Services and Social Welfare, The President’s Office Regional Administration and Local Government, P.O Box 1923, Dodoma, Tanzania; 2 Center for Reforms, Innovation, Health Policies and Implementation Research, Dodoma, Tanzania; 3 Department of Public Health, St Francis University College of Health and Allied Sciences, Ifakara, Tanzania; 4 GE Foundation, Boston, MA USA; 5 Program in Global Surgery and Social Change, Harvard Medical School, Boston, MA USA; 6 Department of Plastic and Oral Surgery, Boston Children’s Hospital, Boston, MA USA; 7 Department of Paediatrics, University of Melbourne, Melbourne, Australia; 8 Department of Anesthesiology and Surgery, Boston Children’s Hospital, Boston, MA USA

**Keywords:** Research capacity, Motivators, Barriers, Engagement, Healthcare providers, Tanzania

## Abstract

**Background:**

Building health research capacity in low- and middle-income countries is essential to achieving universal access to safe, high-quality healthcare. It can enable healthcare workers to conduct locally relevant research and apply findings to strengthen their health delivery systems. However, lack of funding, experience, know-how, and weak research infrastructures hinders their ability. Understanding research capacity, engagement, and contextual factors that either promote or obstruct research efforts by healthcare workers can inform national strategies aimed at building research capacity.

**Methods:**

We used a convergent mixed-methods study design to understand research capacity and research engagement of healthcare workers in Tanzania’s public health system, including the barriers, motivators, and facilitators to conducting research. Our sample included 462 randomly selected healthcare workers from 45 facilities. We conducted surveys and interviews to capture data in five categories: (1) healthcare workers research capacity; (2) research engagement; (3) barriers, motivators, and facilitators; (4) interest in conducting research; and (5) institutional research capacity. We assessed quantitative and qualitative data using frequency and thematic analysis, respectively; we merged the data to identify recurring and unifying concepts.

**Results:**

Respondents reported low experience and confidence in quantitative (34% and 28.7%, respectively) and qualitative research methods (34.5% and 19.6%, respectively). Less than half (44%) of healthcare workers engaged in research. Engagement in research was positively associated with: working at a District Hospital or above (*p* = *0.006*), having a university degree or more (*p* = *0.007*), and previous research experience (*p* = *0.001*); it was negatively associated with female sex (*p* = *0.033*)*.* Barriers to conducting research included lack of research funding, time, skills, opportunities to practice, and research infrastructure. Motivators and facilitators included a desire to address health problems, professional development, and local and international collaborations. Almost all healthcare workers (92%) indicated interest in building their research capacity.

**Conclusion:**

Individual and institutional research capacity and engagement among healthcare workers in Tanzania is low, despite high interest for capacity building. We propose a fourfold pathway for building research capacity in Tanzania through (1) high-quality research training and mentorship; (2) strengthening research infrastructure, funding, and coordination; (3) implementing policies and strategies that stimulate engagement; and (4) strengthening local and international collaborations.

**Supplementary Information:**

The online version contains supplementary material available at 10.1186/s12960-023-00858-w.

## Background

Building and strengthening health research capacity in low- and middle-income countries (LMICs) is essential to achieving universal high-quality and safe health care coverage [1]. Strengthened capacity for research has the potential to nurture hybridization of research and clinical practice, allowing motivated healthcare workers and researchers to generate evidence and apply findings in a locally relevant manner [2, 3]. Nonetheless, lack of financial resources, institutional support and infrastructure, research knowledge and know-how, among many other factors, hinder LMIC-based researchers’ ability to design and implement research projects critical to their needs [4, 5]. Understanding the contextual factors that either promote or obstruct efforts to build research capacity is therefore necessary to inform national strategies aimed to develop strengthened health systems.

Research capacity building is a multi-level process that involves investing in and supporting individuals, teams, organizations, and networks of organizations to increase demand for research, promote researchers’ ability to conduct studies, and enable the effective use of findings [6, 7]. Developing health research capacity is a complex process which involves investing in human, technological, and organizational resources operating at various organizational levels [8, 9]. At the individual level, this includes supporting researchers’ ability to find and critically review literature, generate research ideas, collect and analyze qualitative and quantitative data, write and report results, and find time, mentorship, and funding to conduct research [4, 10–12]. At the facility and systems levels, strengthening research capacity requires increased funding, production of more well-trained investigators, support for regional and international long-term partnerships, along with other administrative improvements in managerial and regulatory mechanisms [13–15]. Because of this complexity, however, numerous barriers hinder the growth of research capacity in LMICs. For instance, a survey of 847 health research institutions in 42 sub-Saharan countries found a significant shortage of well-trained health researchers, a problem which was exacerbated by over-worked individuals who lacked time and motivation [5]. Other identified barriers include high turnover among research staff, inexperience administering research projects, differing expectations among collaborators, competing time demands, limited mentorship, brain drain, difficulty embedding new research activities and success metrics into existing systems, limited regulatory systems and funding, and structural violence and politico-economic instability [4, 16, 17].

Health research in Tanzania has not been spared from these barriers [18]. Tanzania ranks 163 out of 189 countries on the Human Development Index and has a population of about 58 million people [19]. Although a robust research governance structure has been established in the country [20], there remains an urgent need to invest in building and strengthening health research capacity. Assessing the current capacity and identifying existing gaps is a necessary and early component of the change process driving health research capacity building [13]. Therefore, developing a more sophisticated understanding of the barriers, motivators, and facilitators to conducting research is a foundational step in supporting change efforts. This study aims to explore the barriers, motivators, and facilitators experienced by healthcare workers in conducting research, and assess the level of individual and institutional health research capacity and engagement in regional, district, and primary health care facilities in Tanzania.

## Methods

### Study design

We used a convergent mixed-methods study design [21–23] to understand research capacity and engagement and the barriers, facilitators, and motivators to conducting research among healthcare workers in Tanzania. We collected quantitative data from surveys from healthcare workers and their institutions and qualitative data through interviews to triangulate our results and enhance our insights. Together, our analyses provided a more comprehensive understanding of the research landscape in Tanzania. We followed the Good Reporting of a Mixed Methods Study (GRAMMS) framework (Additional file 1) for reporting our results [24].

### Study setting and sample

Our study was conducted in nine geographically dispersed regions, which were randomly selected from the 26 regions of mainland Tanzania. Together, these regions have a total population of 21 119 700, which represents 35.7% of the Tanzanian population. The regions are heterogeneous in population size, distribution of health facilities, distribution of human resources for health, and institutions carrying out health research activities—this provides a comprehensive understanding on research capacity and engagement and their determinants in Tanzania.

Within each of the 9 regions, we selected public health facilities from three levels of the health system—regional, district, and council. First, we selected one urban and one rural council in each of the 9 regions (18 total). Within these, we randomly selected one regional referral hospital, one district hospital, and one health center (45 public health facilities). We also selected two levels of health management teams—1 regional (RHMT) and 2 district (CHMTs) (apart from 3 districts with 1 CHMT) in each region for a total of 9 RHMTs and 15 CHMTs.

**Fig. 1:**
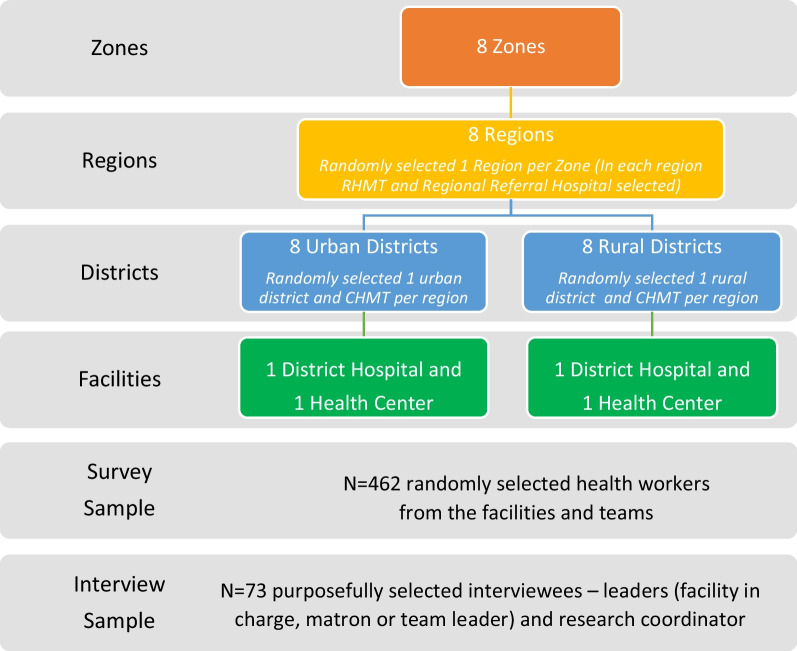
Sampling design

For our quantitative data collection, we randomly selected 462 healthcare workers from the facilities and teams to participate in surveys. For our qualitative data collection, we invited 75 leaders and research coordinators to participate in interview. Leaders were the facility in-charge, or matron or health secretary. Research coordinators were front line workers (e.g., doctor, nurse, nutritionist, laboratory technician), and at the regional level they had additional training in epidemiology, statistics, or public health (Fig. 1).

### Data collection and analysis

#### Survey design

A four-member research team with backgrounds in health services research developed the surveys based on literature on research capacity needs assessments in African settings [5, 25–27] and their experience with Tanzania’s health system. The individual health worker survey questions addressed six topics: (1) research capacity including training, experience, and confidence in research activities; (2) research engagement, type, role, and collaboration; (3) barriers to conducting research; (4) motivators and facilitators for conducting research; (5) interest in conducting research; and (6) respondent characteristics (Additional file 2). The facility survey collected information on two topics: (1) institutional research capacity including connectivity and software, and availability and accessibility of health research resource materials, and (2) facility characteristics (Additional file 3). Responses were either binary (yes/no), selecting from a list, or a 5-point Likert scale. The survey was written in English and translated into Swahili. We pilot-tested the survey with healthcare workers in Dodoma and Chamwino who had similar roles and revised any unclear questions.

#### Quantitative data collection

Surveys were conducted in person by independent, trained research assistants using a Swahili version of the survey on tablets with Open Data Kit software. Research assistants randomly selected healthcare workers in various departments, explained the study and invited their participation. We did not collect any identifiers and no incentives were offered for completing the survey. Electronic data quality checks were conducted daily to ensure data quality and completeness.

#### Qualitative data collection

Interviews were conducted in English and Swahili by independent trained research assistants using a semi-structured interview guide. The interviews explored (1) research engagement; (2) research structures, supports, and processes in place; (3) barriers to conducting research; (4) motivators and facilitators for conducting research; and (5) strengthening the research culture (Additional file 4). The interview protocol was developed in English and translated into Swahili. Interviews were approximately 30 min long and conducted in a private space. Interviews were audio recorded and transcribed verbatim. No participant declined to speak with us or ended the interview prematurely. Transcripts were reviewed for accuracy and uploaded to NVivo V.11 (QSR International, Melbourne, Australia) for coding.

#### Quantitative data analysis

Responses were reported on all survey items in all response categories and summarized using frequencies and percentages. The five response categories on barriers, motivators and facilitators were collapsed into two (none/very small/small/medium and large/very large) and responses to interest in conducting research activities into three (not at all/slightly interested; moderately/50–50 interested; very/extremely interested). We concentrated on the “large/very large” and “very/extremely interested” categories and reported proportions of participants’ responses on selected items, where denominators were the number of responses to the question. We also conducted a descriptive sub-analysis to assess the percentage of respondents that underwent research training by education level and age group. Univariate and multivariable adjusted logistic regression were used to identify the independent factors associated with conducting research. Results were presented as adjusted odds ratios (AOR) with corresponding 95% confidence intervals and *p* values. Statistical significance was defined as a two-sided *p* < *0.05.* Analyses were conducted using SAS software version 9.4 (SAS Institute, Cary, NC) and STATA version 15 (StataCorp LLC. College Station, TX).

#### Qualitative data analysis

Qualitative data were inductively analyzed [23, 28, 29] by three researchers (AK, NZ, SA). First, one researcher AK evaluated 39 transcripts to develop an initial codebook and tested it with 34 different transcripts. Text segments were compared against those previously categorized; codes were refined until no novel codes arose (i.e., code saturation) [30]. Finally, the researcher coded all transcripts and identified recurrent and unifying concepts by connecting and categorizing all codes. Two other members of the research team (NZ and SA) confirmed the validity of the coding manual and the thematic results by coding 20 transcripts.

##### Integrated interpretation

Upon completion of the quantitative and qualitative data analyses, research team members (SA, AK, and NZ) integrated quantitative and qualitative results and identified recurring patterns and themes. Integration occurred at the interpretation level (after completion of data analysis) merging the results and discussing the meaning of the integrated results across the two levels of analysis [31, 32].

### Ethical considerations

Our research protocol was approved by the National Health Research Ethics Review Sub-Committee in Tanzania. Prior to administering the survey or interview participants gave written and informed consent. Participants were informed that their involvement in the study was voluntary and could withdraw at any time for any reason and were provided with the opportunity to ask questions.

## Results

### Respondent characteristics

Table 1 describes the characteristics of 462 survey respondents and the facility or team they represent. The majority of survey respondents were from regional referral hospitals (44.4%), followed by district hospitals (28.1%), health centers (18.8%), and regional and council management teams (4.3% and 4.3%, respectively). Respondents were almost equally female (51.5%) or male (49.5%), just under half had an undergraduate education (45.9%), and one-third of the respondents also had postgraduate qualifications (38.5%). Most were in a clinical position (65.8%), some were in management (18.0%), but only 2 individuals were in a research position (0.4%). Interviewing respondents (*n* = 75) included leaders (medical officer-in-charge, matron, or health secretary) (69.4%) and research coordinators (30.7%) at all facility and team levels.

**Table 1:** Respondent characteristics

Survey respondents (*N* = 462)	*n* (%)
Region	
Dar es salaam	58 (12.6%)
Pwani	41 (8.9%)
Lindi	50 (10.8%)
Tanga	50 (10.8%)
Dodoma	45 (9.7%)
Katavi	50 (10.8%)
Kagera	48 (10.4%)
Kigoma	60 (13.0%)
Njombe	60 (13.0%)
Type of health facility and management team	
Health center	87 (18.8%)
District hospital	130 (28.1%)
Regional referral hospital	205 (44.4%)
Council health management team	20 (4.3%)
Regional health management team	20 (4.3%)
Age	
23–30	119 (25.8%)
31–35	119 (25.8%)
36–40	97 (21.0%)
40+	127 (27.5%)
Sex	
Female	238 (51.5%)
Highest qualification	
Certificate	72 (15.6%)
Undergraduate	212 (45.9%)
Postgraduate	178 (38.5%)
Classification	
Clinical	304 (65.8%)
Management	83 (18.0%)
Clinical education	21 (4.6%)
Research	2 (0.4%)
Other	52 (11.3%)
Occupation	
Specialist	21 (4.6%)
Doctor	81 (17.5%)
Clinical officer	40 (8.7%)
Pharmacist	47 (10.2%)
Laboratory technician	56 (12.1%)
Nurse	137 (29.7%)
Other	80 (17.3%)
Years of work experience	
1–5	248 (53.7%)
6–10	134 (29.0%)
Above 10	80 (17.3%)

Interview respondents (*N* = 75)	
Occupation	
Health secretary	26 (34.7%)
Research coordinator	23 (30.7%)
Facility in-charge/matron	26 (34.7%)
Facility/team level	
Health center	13 (17.3%)
District hospital	19 (25.3%)
Regional referral hospital	13 (17.3%)
Council health management team	13 (17.3%)
Regional health management team	17 (22.7%)
Age	
< 40 years	37 (49.3%)
> 40 years	38 (50.7%)

### Research capacity

#### Individual healthcare worker level

Table 2 shows the research capacity of individual healthcare workers. Over half of the respondents had undergone research training (59.7%), typically at a university or medical college (81.2%). The majority of respondents with certificate-level education did not receive research training. For those with an undergraduate degree, more respondents in the 23–30 year and over 40 years age categories had research training. For those with postgraduate degrees, more respondents over the age of 36 years had research training (Additional file 7).

**Table 2:** Research capacity of healthcare workers

Variable	*n* (%)
Research capacity (*N* = 462)	
Ever undergone research training	
Yes	276 (59.74%)
Where training was received*	
University or medical college	224 (81.2%)
Professional development training	36 (13.0%)
Work experience	13 (4.7%)
Other	3 (1.1%)
Research identified in job description	
Yes	162 (35.1%)

^*^N = 276

Respondents reported low experience and confidence in quantitative (34% and 28.7%, respectively) and qualitative research methods (34.5% and 19.6%, respectively), applying for funding (12% and 7.7%, respectively), analyzing and interpreting results (28.2% and 22.5%, respectively), and presenting (21.5% and 16.8%, respectively) or publishing results (18.7% and 10.1%, respectively) (Additional file 5).

#### Facility level

Table 3 shows research capacity at the institutional level. Two-thirds (66.1%) of facilities provided free access to internet. Of those facilities, the majority (53.2%) restricted access to senior and middle management, or technical staff with specific duties. Similarly, computers were provided mainly to senior and middle management (69.3% and 83.7%, respectively). Almost three-quarters of facilities (71%) did not have statistical packages. Only 17.7% had access to free electronic journals, 11.3% received access to HINARI (a program to provide free or low-cost online access to journals) [33], and 3.2% had a library. Approximately one-third of the facilities had a research coordinator (38.7%).

**Table 3:** Connectivity and software, and availability and accessibility of health research resource materials

Variable	*n* (%)
Connectivity and software in facilities (*N* = 62)	
Networks and support	
IT support locally stationed	28 (45.2%)
IT support available if needed	18 (29%)
No IT support	16 (25.8%)
Access to internet	
Daily access and paid by the organization	41 (66.1%)
Available but cost covered by individuals	8 (12.9%)
No internet access	13 (21%)
Statistical packages	
Not available	44 (71%)
Available but owned by employee	11 (17.7%)
Provided by the institution and easily accessible	3 (4.8%)
Provided by the institution but not easily accessible	4 (6.5%)
Provision of computer	
All staff	12 (19.4%)
Only for leaders	11 (17.7%)
Middle level management	32 (51.6%)
Does not provide computer	7 (11.3%)
Provision of printer	
All staff	8 (14.6%)
Only for leaders	14 (25.5%)
Middle level management	32 (58.2%)
Does not provide computer printer	1 (1.8%)
Provision of internet access	
All staff	19 (30.7%)
Only for leaders	2 (3.2%)
Middle level management	21 (33.9%)
Provided to technical staff with specific duties	10 (16.1%)
Does not provide internet access	10 (16.1%)
Availability and accessibility of health research resources materials (*N* = 62)	
Access to HINARI	7 (11.3%)
Access to free electronic journals	11 (17.7%)
Accessibility of hard copies of scientific journals	22 (35.5%)
Availability of a library	2 (3.2%)
Availability and accessibility of books	25 (40.3%)
Having a research coordinator	24 (38.7%)

### Research engagement

Table 4 shows the research engagement of healthcare workers. Less than half of the respondents (44.2%) reported ever conducting research. Of those respondents, approximately one-third had experience with clinical trials (35.3%), health services research (32.8%), and behavioral or sociological research (32.4%). Fewer had experience with epidemiological research (19.1%) and health system and policy research (13.7%). Research was usually conducted independently (59.8%), but some participants engaged in collaboration with local universities (37.8%), local and international NGOs (31.7%, 25.6%), local research institutions (24.4%), or the Tanzania National Medical Research Institution (18.3%).

**Table 4:** Research engagement of healthcare workers

Variable	*n* (%)
Research engagement (*N* = 204)	
Ever conducted research	
Yes	204 (44.2%)
Type of research conducted	
Health system and policy-related research	28 (13.7%)
Health services research other than clinical trials	67 (32.8%)
Behavioral or sociological research	66 (32.4%)
Clinical trials	72 (35.3%)
Epidemiological research	39 (19.1%)
Research role	
Research assistant	82 (40.2%)
Principal investigator	98 (48.0%)
Co-principal investigator	12 (5.9%)
Policy advisor	1 (0.5%)
Other	11 (5.4%)
Independent or collaborative research	
Independent	122 (59.8%)
Collaborative	82 (40.2%)
Collaborators	
Local University	31 (37.8%)
International University	1 (1.2%)
Local NGOs	26 (31.7%)
International NGOs	21 (25.6%)
Local Research Institution	20 (24.4%)
National Institute for Medical Research	15 (18.3%)

### Barriers, facilitators, and motivators for research engagement

Below we present quantitative and qualitative results on barriers, facilitators, and motivators to conducting research. We describe the themes emerging from qualitative interviews at three levels: (individual) capability, organizational, and environmental. Illustrative quotes are presented, and have been edited for conciseness.

#### Barriers

**Fig. 2:**
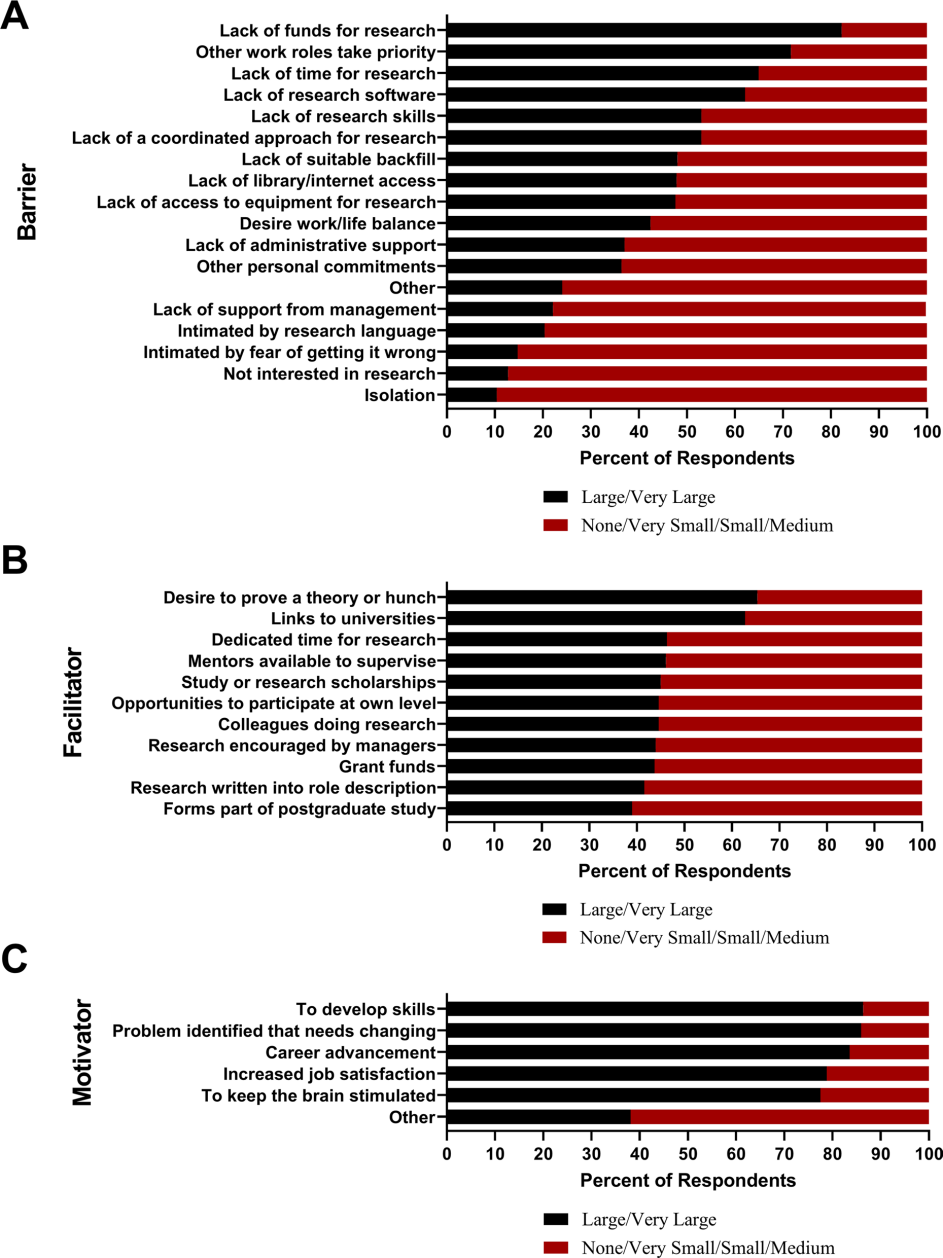
Barriers, facilitators and motivators to conducting research among healthcare workers in Tanzanian public health facilities

The top five barriers to conducting research reported by respondents were: lack of research funding (82.3%), clinical duties taking priority over research (71.7%), lack of time (64.9%), lack of research software (62.1%), and lack of research skills among healthcare workers (53%) (Fig. 2a).

##### Capability barriers

A key barrier reported in interviews was the lack of research skills that would allow participants to engage in research projects. While many had received some research training through their education, there have been few research opportunities to utilize those skills since. One participant described the issue of dormant research skills:

*Another issue is knowledge on research…I can only recall pieces of information from my diploma studies, I have not been trained while on work, and therefore this is something new for me. (Medical officer-in-charge, Health Center)*

##### Organizational barriers

Participants noted several organizational barriers at the staff level. They described being overburdened with many clinical and administrative responsibilities that left them with little to no time for research. Additionally, low monetary compensation, no protected time for research, and a lack of sufficient staff to distribute clinical duties reduced motivation.

Participants noted the lack of basic research infrastructure in their facilities as an additional barrier. Internet, computers, or journals were often not available to them, which did not facilitate easy data collection. Only a few facilities had a designated research coordinator or data manager, and none had a designated research department. The absence of research meetings, forums, and opportunities to travel also limited engagement. One participant explained how organizational barriers impeded research:

*We have to conduct research to reduce disease outbreaks but we are just making sure that medicines are available and patients are served well. We do not have time to conduct research to see why these diseases are there and how we can reduce the rate. We have shortage of staff, time, and funds and we just use the data we have to provide medical assistance in health facilities without addressing how to reduce the rate of disease outbreaks in the community. (Health Secretary, CHMT*)

##### Environmental barriers

Participants noted that regulatory mechanisms for research, such as obtaining ethical clearance, were complicated. Additionally, lack of research funding hindered research. Budgets were focused on clinical priorities; healthcare workers lacked both time and skills to develop research proposals to attract external funding. When local or external research groups had funding, they either utilized healthcare workers to collect data or perform coordination tasks or did not involve them entirely. One participant explained:

*Basically, we are involved in research activities that have been initiated by upper levels but as a team we do not initiate any research activities. So we are just participants in others research mainly implemented by international donor agencies/partners in our settings. (District Health Secretary, CHMT)*

Participants explained that the capability, organizational, and environmental barriers did not foster a culture of research and contributed to low levels of research.

#### Facilitators

Participants identified facilitators that helped them engage in research despite barriers. The top five facilitators to conducting research were: desire to prove a theory (46.3%), connections to universities (62.8%), time dedicated for research (46.3%), having mentors to guide research initiatives (46.1%), and scholarships to support research endeavors (45%) (Fig. 2b).

##### Capability facilitators

Some participants mentioned understanding the value of research in improving knowledge to effectively address health problems in their community. In some facilities, participants mentioned having healthcare workers with research skills and experience who could lead research activities. Furthermore, some (regional) facilities and CHMTs and RHMTs had a dedicated research staff. One participant explained:

*As a team we have an epidemiologist, statistician, and data officer. Management committees also exist. So it is possible to successfully conduct research because all these individuals may significantly help to execute any research. (District Health Secretary, CHMT)*

##### Organizational facilitators

Participants reported that routine data collection provided opportunities to conduct research. Additionally, having a data manager or a research coordinator gave facilities the opportunity to engage in research projects. Facilities and teams also had committees which could approve research projects. One participant explained:

*The fact that we have plenty of data at the hospital, we are motivated to conduct research, and sometimes through complaints and opinions from our patients (Matron, Regional Referral Hospital)*

A few participants noted that their facility had begun to designate a budget specifically for research; while this could not fund all activities, it allowed for the development of a research culture.

##### Environmental context and resource facilitators

Participants reported that external support by development partners and collaborators from universities and research institutions facilitated health research. They identified the need for funding from local and external institutions and the importance of working with healthcare workers to conduct research. Additionally, participants noted that they received encouragement from governmental entities (i.e., the Ministry of Health) to conduct research initiatives.

#### Motivators

The top five motivators for conducting research are: a desire to develop research skills (86.4%), identifying clinical problems and wanting to understand and change them through research (85.9%), a desire to advance one’s own career (83.6%), improving job satisfaction (78.8%), and keeping the brain stimulated with new challenges (77.5%) (Fig. 2c).

In interviews, three major themes emerged. First, almost all participants expressed a desire to understand the causes for poor health outcomes—they wanted evidence-based solutions to improve patient outcomes. Second, they wanted to improve the organization and provision of health services. Third, they were motivated to develop their research skills. One participant explained their motivation for engaging in research:

*The challenges I have been facing in my work, nursing care plan does not go as expected that I saw there is a need to find out the cause and come up with the solution, and also to increase my personal skills. (Matron, Regional Referral Hospital)*

### Opportunities to improve research engagement

Participants provided suggestions on how engagement in research could be improved in their facilities. Participants highlighted four ideas: (1) developing research skills through in-person courses and research mentorship; (2) financial and technological support from entities such as the Ministry of Health; (3) increasing budgets to hire more staff to share the clinical workload and establish a dedicated research team; and (4) collaborating with local and international partners. Participants emphasized that addressing all these components would establish a receptive climate and provide greater motivation for long-term research engagement.

### Building future research capacity

The vast majority (92%) of Tanzanian healthcare workers surveyed indicated an interest in building their research capacity. The top priorities for research capacity building are: learning how to apply for funding (82%), gaining skills to write and publish papers (81%), managing a project (80%), learning how to write and present abstracts (75%), and gaining skills to analyze and interpret data (74%) (Additional file 6).

### Regression model on factors influencing engagement in research

Table 5 presents the results for the final adjusted multivariable logistic regression model (c-statistic = 0.898). Four factors were significant independent predictors of an increased odds of involvement in research: working in a district hospital (*p* = 0.006) or a regional or council health management team (*p* = 0.024); having an undergraduate qualification (*p* = 0.007), having a postgraduate qualification (*p* = 0.014); age ≥ 40 (*p* = 0.034) and having prior experience in research process activities (*p* < 0.001). Prior experience with research was associated with a 25-fold increase in the odds of reporting involvement in research (AOR = 22.82, CI = (12.57–41.40), *p* < 0.001). One factor was independently associated with a decreased likelihood involvement in research: female gender (*p* = 0.033).

**Table 5:** Binary logistic analysis for factors associated with involvement in research

Variable	Uninvolved	Involved	Unadjusted analysis		Adjusted analysis	
	*n* (%)	*n* (%)	OR [95% CI]	*p*-value	AOR [95% CI]	*p*-value
Type of health facility						
Health center	66 (75.9%)	21 (24.1%)	Ref.		Ref.	
District hospital	80 (61.5%)	50 (38.5%)	1.96 [1.07, 3.59]	0.029	3.18 [1.39, 7.28]	0.006
Regional ref. hospital	94 (45.8%)	111 (54.2%)	3.71 [2.11, 6.52]	< 0.001	2.41 [1.12, 5.18]	0.024
RS/RHMT/CHMT	18 (45%)	22 (55%)	3.84 [1.74, 8.4]	< 0.001	3.71 [0.99, 13.81]	0.051
Age (years)						
23–30	72 (60.5%)	47 (39.5%)	Ref.		Ref.	
31–35	75 (63%)	44 (37%)	0.90 [0.53, 1.52]	0.689	0.92 [0.41, 2.06]	0.848
36–40	57 (58.8%)	40 (41.24%)	1.08 [0.62, 1.86]	0.795	1.50 [0.61, 3.71]	0.375
40+	54 (42.5%)	73 (57.5%)	2.07 [1.25, 3.44]	0.005	2.92 [1.09, 7.79]	0.034
Sex						
Male	104 (46.4%)	120 (53.6%)	Ref.		Ref.	
Female	154 (64.7%)	84 (35.3%)	0.47 [0.33, 0.69]	< 0.001	0.55 [0.31, 0.96]	0.033
Highest qualification						
Certificate	67 (93.1%)	5 (6.9%)	Ref.		Ref.	
Undergraduate	114 (53.8%)	98 (46.2%)	11.51 [4.46, 29.72]	< 0.001	5.13 [1.57, 16.74]	0.007
Postgraduate	77 (43.3%)	101 (56.7%)	17.57 [6.76, 45.70]	< 0.001	4.65 [1.36, 15.85]	0.014
Classification level of your current position						
Clinical	167 (54.9%)	137 (45.1%)	Ref.		Ref.	
Management	50 (48.1%)	54 (51.9%)	1.32 [0.84, 2.06]	0.227	0.74 [0.36, 1.53]	0.426
Others	41 (75.9%)	13 (24.1%)	0.39 [0.20, 0.75]	0.005	0.35 [0.12, 0.99]	0.048
Cadre						
Clinical officer	28 (70%)	12 (30%)	Ref.		Ref.	
Doctor	39 (38.2%)	63 (61.8%)	3.77 [1.72, 8.26]	< 0.001	1.12 [0.39, 3.25]	0.832
Pharmacist	26 (55.3%)	21 (44.7%)	1.88 [0.78, 4.58]	0.162	1.46 [0.46, 4.62]	0.523
Laboratory technician	27 (48.2%)	29 (51.8%)	2.51 [1.07, 5.90]	0.035	2.19 [0.70, 6.89]	0.180
Nurse	89 (65%)	48 (35%)	1.26 [0.59, 2.69]	0.555	1.63 [0.58, 4.56]	0.350
Other	49 (61.3%)	31 (38.8%)	1.48 [0.66, 3.33]	0.348	0.98 [0.30, 3.25]	0.976
Years of experience						
1 to 5	151 (60.5%)	97 (39.1%)	Ref.		Ref.	
6 to 10	69 (51.5%)	65 (48.5%)	1.47 [0.96, 2.24]	0.077	1.11 [0.57, 2.18]	0.763
10+	38 (47.5%)	42 (52.5%)	1.72 [1.04, 2.86]	0.036	0.58 [0.23, 1.47]	0.255
Ever undergone training in research						
No	141 (75.8%)	45 (24.2%)	Ref.		Ref.	
Yes	117 (42.4%)	159 (57.6%)	4.26 [2.82, 6.43]	< 0.001	1.17 [0.61, 2.25]	0.629
Having research tasks in job description						
No	187 (62.3%)	113 (37.7%)	Ref.		Ref.	
Yes	71 (43.8%)	91 (56.2%)	2.12 [1.44, 3.13]	< 0.001	1.05 [0.58, 1.90]	0.862
Having experience in engaging in the research process activities						
No	216 (85.4%)	37 (14.6%)	Ref.		Ref.	
Yes	42 (20.1%)	167 (79.9%)	23.21 [14.28, 37.7]	< 0.001	22.82 [12.57, 41.40]	< 0.001

## Discussion

This study represents the first comprehensive evaluation of research capacity among healthcare workers in Tanzania. Our findings reveal that research capacity and engagement are low among healthcare workers in Tanzania, while also highlighting a strong interest in research participation. We found that research engagement is positively associated with place of employment, having a degree, age over 40 years, and previous research experience; it is negatively associated with identifying as female. We found several barriers to research, including lack of funding, time, skills, opportunities to practice, and research infrastructure. On the other hand, motivators and facilitators of research included a desire to address health problems, professional development, and support from local and international collaborators.

Our findings are consistent with other studies from LMICs. In particular, research capacity was reported to be low in other African countries [27, 34, 35] and in Pakistan [35]. Additionally, participation in research has been linked to individuals' education level and previous research experience [36, 37] and women were less likely to participate in research [36].

We found that lack of research funding was a significant barrier to conducting research in Tanzania, consistent with evidence from other African settings [34, 37–43]. However, other factors may contribute to the challenges of accessing research funding, including inadequate skills and knowledge in developing competitive grant proposals [44, 45], absence of dedicated time for research activities [40, 46], and low access to internet and library facilities [27]. Interestingly, despite the availability of free statistical programs and electronic journals, these resources are often underutilized, possibly due to a lack of awareness [47], technology barriers, and insufficient expertise to access them [48]. Furthermore, time limitations to participate in research have been well-documented in other LMICs [25, 37, 41, 49], as well as some high-income countries [25, 50, 51].

Mentors, as well as local and international collaborations emerged as facilitators of engagement in research in our study, which is consistent with other studies on the topic [17, 39, 52–54]. Additionally, the desire to conduct research to solve healthcare challenges and improve patient outcomes emerged as a key motivator in our quantitative and qualitative, in line with evidence from both low- and high-income countries [25, 37, 39, 40, 49].

Improving research capacity among healthcare workers in Tanzania is essential to generate practical, innovative, local solutions for improving health quality and systems [34, 55], advancing Universal Health Coverage [1], and promoting economic transformation [10]. Our findings suggest that building research capacity in LMICs requires a multifaceted approach for success. We propose a fourfold pathway for building research capacity.

First, it is critical to focus on building the capacity of individual healthcare workers through high-quality training [26, 53], setting the national research agenda [35], and collaboration with academic institutions for cost-efficient trainings and sharing of expertise [10, 56]. Despite 84.4% of the sample having a degree, only 59.74% reported receiving research training, highlighting the need for educational policy interventions to bridge this gap. An in-depth analysis of curricula taught at the undergraduate and postgraduate education levels can provide objective data to guide policy interventions. It is also essential to target research training towards all healthcare workers including doctors [57], nurses [57], pharmacists [58], and other allied health professionals. Interprofessional collaboration in conducting research is crucial to benefit patients, as the different cadres are dependent on each other [59]. Future studies could assess current capacity and interest in various fields, methodologies, and types of research along the basic, translational, clinical, and implementation sciences to develop a responsive training intervention. Additionally, mentorship has been shown to influence personal and professional development and research productivity [60], therefore, creating a local pool of mentors can provide opportunities for healthcare workers to continue with their work while receiving the necessary guidance and support [10].

Second, research infrastructure and funding are essential to fostering an enabling research environment. Internet, computers, printers, and journals are necessary to build a research infrastructure. Promoting awareness and building capacity in researchers to use free electronic journals and statistical programs will further improve participation in research [34, 34]. Additionally, empowering researchers with skills and knowledge in identifying funding opportunities, preparing grant proposals, and networking between research teams may enable effective and efficient use of resources and increase chances of obtaining competitive research grants [10].

Third, it is crucial to implement policies and strategies that facilitate a supportive research environment. Policymakers can foster such an environment by strengthening data infrastructure, promoting routine data use to support decision-making, appointing dedicated research coordinators, linking promotion to research participation, providing avenue for sharing frontline workers research and recognition, integrating research agenda in work place, requiring local and international researchers to collaborate with healthcare workers in facilities, promoting adjunct research fellowship or attachment in research institutions or universities and empowering women to participate in research activities.

Finally, strengthening both local and international collaborations for research is essential in skills building and empowering frontline workers’ capability for conducting research. Studies have shown that international collaboration in health research brings about many benefits, including opportunities for knowledge transfer, expertise sharing, and increased funding [17, 53]. Collaborations also enable joint participation in problem identification, research proposal development, research execution, publication, and the establishment of a community of practice. Moreover, they promote continuous learning, generate knowledge to support the design of interventions and policies, and improve services, infrastructure, and the availability of financial resources [61].

## Strengths and limitations

This study has a number of strengths. It is the first comprehensive study of research capacity, engagement, motivators, and barriers for conducting research in Tanzania, and covers a large, representative sample using a concurrent mixed-methods approach. However, this study is limited in that it is a cross-sectional study, hence associations are not causal. Our survey had some limitations. We did not include a clear definition of research and its scope. This may have resulted in different interpretations of questions by various cadres of healthcare workers. Furthermore, the survey did not collect detailed information on the type of research training received, which could have provided more insights. Moreover, the study did not collect data on the quantity and quality of research, which would have been useful in assessing research productivity and quality. Finally, the qualitative interviews may have been susceptible to social desirability bias.

## Conclusions

Research is key to improving health outcomes, however, research capacity is low in Tanzania’s public health facilities. Healthcare workers in Tanzania are highly interested in engaging in research, despite individual and institutional capability gaps. We propose a pathway for building research capacity in Tanzania through: developing and implementing high-quality and tailored research training programs and strong mentorship, strengthening the health research infrastructure and funding, implementing policies and strategies that stimulate engagement in research activities, and strengthening local and international collaborations for research.

### Supplementary Information

**Additional file 1.** GRAMMS framework—checklist.

**Additional file 2.** Individual health worker survey.

**Additional file 3.** Facility survey.

**Additional file 4.** Interview protocol.

**Additional file 5.** Experience and confidence in conducting research activities.

**Additional file 6.** Priorities for developing future research capacity.

**Additional file 7.** Percentage of respondents who reported receiving research training by education level and age group.

## Data Availability

The datasets used and/or analyzed during the current study are available from the corresponding author on reasonable request.

## Abbreviations

CHMT: Council health management team
LMICs: Low- and middle-income countries
RHMT: Regional health management team
