# Supplementary material for: Research capacity, motivators and barriers to conducting research among healthcare providers in Tanzania’s public health system: a mixed methods study
Source: Hum Resour Health. 2023 Sep 5;21:73. doi: 10.1186/s12960-023-00858-w (PMC10478476; doi:10.1186/s12960-023-00858-w)
Supplement: Supplementary file 1 — Additional file 1. GRAMMS framework—checklist. [file 12960_2023_858_MOESM1_ESM.doc]

**Additional file 1**: GRAMMS framework—Checklist of items that should be included in **mixed methods studies**

| Item No | Recommendation | Reported on page |
| --- | --- | --- |
| 1 | Describe the justification for using a mixed methods approach to the research question | 8 |
| 2 | Describe the design in terms of the purpose, priority and sequence of methods | 8 |
| 3 | Describe each method in terms of sampling, data collection and analysis | 8-11 |
| 4 | Describe where integration has occurred, how it has occurred and who has participated in it | 12 |
| 5 | Describe any limitation of one method associated with the presence of the other method | 23 |
| 6 | Describe any insights gained from mixing or integrating methods | 23 |
