# Supplementary material for: Research capacity, motivators and barriers to conducting research among healthcare providers in Tanzania’s public health system: a mixed methods study
Source: Hum Resour Health. 2023 Sep 5;21:73. doi: 10.1186/s12960-023-00858-w (PMC10478476; doi:10.1186/s12960-023-00858-w)
Supplement: Supplementary file 2 — Additional file 2. Individual health worker survey. [file 12960_2023_858_MOESM2_ESM.doc]

**Additional file 2**. Individual health worker survey

|  | **Question** | **Response** |
| --- | --- | --- |
| *Section 1: Background Information* | | |
| 1a | Questionnaire number |  |
| 1b | Code of interviewer |  |
| 1c | Name of the region |  |
| 1d | Name of the Council |  |
| 1e | Name of Health Facility/CHMT/RHMT |  |
| 1f | Type of health facility | - Health center - District/council hospital - Regional hospital - RS/RHMT - CHMT - Other (specify) |
| 1g | Does this facility act as a higher learning teaching hospital? | Circle one:  Yes No |
| *Section 2: Participant Socio-Demographic Information* | | |
| 2a | Sex | - Female - Male |
| 2b | Age (years) |  |
| 2c | What is your highest qualification? | - Certificate - Undergraduate - Postgraduate - Doctor of Philosophy - Professional doctorate degree - Professional and research doctorate degree - Nil |
| 2d | What is the classification level of your current position? | - Clinical - Management - Clinical education - Research |
| 2e | How many years have you worked in this role at any health facility? |  |
| 2f | What is your employment status? | - Permanent - Locum/temporary/fixed contract |
| *Section 3: Research Engagement/Participation* | | |
| 3a | Have you ever undergone training in research? If yes, where? | - As part of my formal training in the university or medical college - As part of Continuous Professional Training after been employed - Through project support in our institution - Other (specify) |
| 3b | Do you have research tasks in your role (job) description? | - Yes - No |
| 3c | Have you ever conducted research? | - Yes (If yes, go to the next question. If no, skip the next question) - No |
| 3d | What types of research were you involved in (multiple responses)? | - Health system and policy-related research - Health services research other than clinical trials (e.g., monitoring and evaluation of health services). - Behavioral or sociological research (e.g., substance use among urban youth). - Clinical trials (e.g., testing patient safety and efficacy of a new drug). - Epidemiological research (e.g. Outbreak investigations) |
| 3e | If yes above, what roles have you participated in? | - Research Assistant - Principal Investigator - Co-Principal investigator - Policy advisor - Others (specify) |
| 3f | Do you conduct research as independent or in collaboration (If with collaboration; Please select the appropriate option) | - Collaborate with Local University - Collaborate with International University - Collaborate with local NGOs - Collaborate with International NGOs - Collaborate with local research institution such as (IHI, KCRI and MITU e.t.c) - Collaborate with National Institute for Medical Research - Others (specify) |
| *Section 4: Interest, experience, confidence, and priority* | | |
|  |  |  |

*Section 4: Interest, experience, confidence, and priority*

**4a. On a scale of 0 to 5 indicate your area of interest in research capacity building for the following items. 0 means the least rated in interest, and 5 means the highest rated in interest.**

|  | **Area of Interest** | **0** | **1** | **2** | **3** | **4** | **5** |
| --- | --- | --- | --- | --- | --- | --- | --- |
| 1 | Finding relevant literature |  |  |  |  |  |  |
| 2 | Critically reviewing literature |  |  |  |  |  |  |
| 3 | Generating research ideas |  |  |  |  |  |  |
| 4 | Writing a research proposal |  |  |  |  |  |  |
| 5 | Research ethics |  |  |  |  |  |  |
| 6 | Using research software (e.g. SAS, STATA, Nvivo, etc.) |  |  |  |  |  |  |
| 7 | Using quantitative research methods |  |  |  |  |  |  |
| 8 | Using qualitative research methods |  |  |  |  |  |  |
| 9 | Using mixed methods research design |  |  |  |  |  |  |
| 10 | Applying for research funding |  |  |  |  |  |  |
| 11 | Analyzing and interpreting results |  |  |  |  |  |  |
| 12 | Writing and presenting abstract or paper |  |  |  |  |  |  |
| 13 | Writing and publishing research |  |  |  |  |  |  |
| 14 | Managing a research project |  |  |  |  |  |  |

**4b. What research areas do you currently have experience in?**

- Finding relevant literature
- Critically reviewing literature
- Generating research ideas
- Writing a research proposal
- Research ethics
- Using research software (e.g. SAS, STATA, Nvivo, etc.)
- Using quantitative research methods
- Using qualitative research methods
- Using mixed methods research design
- Applying for research funding
- Analyzing and interpreting results
- Writing and presenting abstract or paper
- Writing and publishing research
- Managing a research project

**4c. Which research areas do you feel confident in?**

- Finding relevant literature
- Critically reviewing literature
- Generating research ideas
- Writing a research proposal
- Research ethics
- Using research software (e.g. SAS, STATA, Nvivo, etc.)
- Using quantitative research methods
- Using qualitative research methods
- Using mixed methods research design
- Applying for research funding
- Analyzing and interpreting results
- Writing and presenting abstract or paper
- Writing and publishing research
- Managing a research project

**4d. On a scale of 0-5 for the following items indicate priority areas for research training (Consider a situation of limited resources). 0 being least rated as a priority and 5 being highly rated as a priority.**

|  | **Area of Interest** | **0** | **1** | **2** | **3** | **4** | **5** |
| --- | --- | --- | --- | --- | --- | --- | --- |
| 1 | Finding relevant literature |  |  |  |  |  |  |
| 2 | Critically reviewing literature |  |  |  |  |  |  |
| 3 | Generating research ideas |  |  |  |  |  |  |
| 4 | Writing a research proposal |  |  |  |  |  |  |
| 5 | Research ethics |  |  |  |  |  |  |
| 6 | Using research software (e.g. SAS, STATA, Nvivo, etc.) |  |  |  |  |  |  |
| 7 | Using quantitative research methods |  |  |  |  |  |  |
| 8 | Using qualitative research methods |  |  |  |  |  |  |
| 9 | Using mixed methods research design |  |  |  |  |  |  |
| 10 | Applying for research funding |  |  |  |  |  |  |
| 11 | Analyzing and interpreting results |  |  |  |  |  |  |
| 12 | Writing and presenting abstract or paper |  |  |  |  |  |  |
| 13 | Writing and publishing research |  |  |  |  |  |  |
| 14 | Managing a research project |  |  |  |  |  |  |

Section 5: Motivators, enablers and barriers for researchers at an individual level

**5a. On a scale of 0 to 5 indicate how each of the following barriers of research apply to you personally? 0 is least rated as a barrier, and 5 is highly rated as a barrier.**

|  | **Barriers for conducting research** | **0** | **1** | **2** | **3** | **4** | **5** |  |
| --- | --- | --- | --- | --- | --- | --- | --- | --- |
| 1 | Other work roles take priority |  |  |  |  |  |  |  |
| 2 | Lack of time to do research |  |  |  |  |  |  |  |
| 3 | Desire for work/life balance |  |  |  |  |  |  |  |
| 4 | Lack of funds for research |  |  |  |  |  |  |  |
| 5 | Lack of skills for research |  |  |  |  |  |  |  |
| 6 | Lack of suitable backfill |  |  |  |  |  |  |  |
| 7 | Lack of administrative support |  |  |  |  |  |  |  |
| 8 | Lack of software for research |  |  |  |  |  |  |  |
| 9 | Lack of a coordinated approach for research |  |  |  |  |  |  |  |
| 10 | Other personal commitments |  |  |  |  |  |  |  |
| 11 | Lack access to equipment for research  results |  |  |  |  |  |  |  |
| 12 | Intimated by research language |  |  |  |  |  |  |  |
| 13 | Intimated by fear of getting it wrong |  |  |  |  |  |  |  |
| 14 | Lack of support from management |  |  |  |  |  |  |  |
| 15 | Not interested in research |  |  |  |  |  |  |  |
| 16 | Isolation |  |  |  |  |  |  |  |
| 17 | Lack of library/internet access |  |  |  |  |  |  |  |
| 18 | Other (e.g. limited exposure to research, lack of access to expertise, statistical analysis, lack of knowledge) |  |  |  |  |  |  |  |

**5b. In a Scale of 0 to 5 indicate how each of the following motivators to do research apply to you personally? 0 is least rated as a motivator, and 5 is highly rated as a motivator.**

|  | **Motivators for conducting research** | **0** | **1** | **2** | **3** | **4** | **5** |
| --- | --- | --- | --- | --- | --- | --- | --- |
| 1 | To develop skills |  |  |  |  |  |  |
| 2 | Increased job satisfaction |  |  |  |  |  |  |
| 3 | Problem identified that needs changing |  |  |  |  |  |  |
| 4 | To keep the brain stimulated |  |  |  |  |  |  |
| 5 | Career advancement |  |  |  |  |  |  |
| 6 | Research encouraged by managers |  |  |  |  |  |  |
| 7 | Links to universities |  |  |  |  |  |  |
| 8 | Mentors available to supervise |  |  |  |  |  |  |
| 9 | Opportunities to participate at own level |  |  |  |  |  |  |
| 10 | Desire to prove a theory or hunch |  |  |  |  |  |  |
| 11 | Dedicated time for research |  |  |  |  |  |  |
| 12 | Colleagues doing research |  |  |  |  |  |  |
| 13 | Grant funds |  |  |  |  |  |  |
| 14 | Forms part of postgraduate study |  |  |  |  |  |  |
| 15 | Research written into role description |  |  |  |  |  |  |
| 16 | Study or research scholarships |  |  |  |  |  |  |
| 17 | Other (e.g. to gather evidence that is relevant to practice, to increase knowledge, to keep at cutting edge, support a new health initiative) |  |  |  |  |  |  |

Section 6: Elements of a successful program

**6a. What is your motivation to do research?**

- To improve the quality of care
- To use data for policy or decision making
- To bring in additional resources
- To develop programs
- To evaluate the effectiveness of programs
- To advance my career

Section 7: Elements of effective trainings

**7a. How much time would you be willing to dedicate to a research-training program?**

- One full day per week, over several months
- One full day every 2 weeks, over several months
- One full day per month, over several months
- Two or 3 full-day intensive
- One-week intensive

**7b. On a scale of 0-5 how effective do you think the following methods of training would be in achieving your learning goals? 0 is least rated as effective and 5 is highly rated as effective.**

|  | **Motivators for conducting research** | **0** | **1** | **2** | **3** | **4** | **5** |
| --- | --- | --- | --- | --- | --- | --- | --- |
| 1 | Workshops/classroom setting |  |  |  |  |  |  |
| 2 | Webinar/Zoom |  |  |  |  |  |  |
| 3 | Toolkit/resource material |  |  |  |  |  |  |
| 4 | Mentorship |  |  |  |  |  |  |
| 5 | On the job training |  |  |  |  |  |  |
