# Supplementary material for: Research capacity, motivators and barriers to conducting research among healthcare providers in Tanzania’s public health system: a mixed methods study
Source: Hum Resour Health. 2023 Sep 5;21:73. doi: 10.1186/s12960-023-00858-w (PMC10478476; doi:10.1186/s12960-023-00858-w)
Supplement: Supplementary file 3 — Additional file 3. Facility survey. [file 12960_2023_858_MOESM3_ESM.doc]

**Additional File 3**. Facility survey

**Section 1. Background information**

1. Please identify the characteristics of your Organization/Team (RS/RHMT/CHMT) in the following areas: -
   1. Ownership
      1. Public
      2. Private not-for-profit (e.g., Faith based organization)
   2. Type of Organization/Team
      1. Health Centre
      2. District/Council Hospital/DDH
      3. Regional Referral Hospital
      4. RS/ RHMT
      5. CHMT
   3. Location of the facility/team
      1. Rural
      2. Urban
      3. Semi-Urban
   4. How many employees exist in your organization or team…...…….?
   5. How many personnel are involved in research in your organization …………………?
   6. How much in your Annual budget is allocated for research activities ………………… (Tshs)?
   7. What is the source of fund for research activities?
      1. Own source
      2. HSBF
      3. O.C
      4. GF
      5. Implementing partners
      6. Other (Specify)

**Section 2. Health research infrastructure and coordination**

1. What is the current research infrastructure in your organization ***(Please check the most appropriate answer in the following items)***
2. Connectivity and software

A1) Networks and support

- - - 1. IT support locally stationed
      2. IT support available if needed
      3. No IT supports

A2) Access to internet

1. Daily access to internet paid by the organization
2. Available but, cost covered by individuals
3. No internet accesses

A3) Statistical packages (SPSS, Stata etc.)

1. Not available
2. Available but personal (owned by employee)
3. Provided by the institution and easily accessible
4. Provided by the institution but not easily accessible
5. Access to computers and IT service

B1) Provision of computer

- - - 1. All staff
      2. Only for leaders (Top level management)
      3. Middle level management (Head of Departments/Section or units)
      4. Does not provide computer

B2) Provision of computer printer (access to)

1. All staff
2. Only for leaders (Top level management)
3. Middle level management (Head of Departments/Sections or units)
4. Does not provide computer printer

B3) Provision of internet access

1. All staff
2. Only for leaders (Top level management)
3. Middle level management
4. Provided to technical staff with specific duties (e.g. HMIS focal person)
5. Does not provide internet access
6. Availability and accessibility of health research resources materials
   - 1. Access to HINARI
     2. Access to free electronic journals
     3. Hard copies of current scientific journals
     4. Books
7. Does your facility/Team have a research coordinator (Yes/No)
8. If yes, how do you rate in a scale of 0 to 5 the following coordination activities in your organization/institution?

|  | **Motivators for conducting research** | **0** | **1** | **2** | **3** | **4** | **5** |
| --- | --- | --- | --- | --- | --- | --- | --- |
| 1 | How do you rate coordination activities |  |  |  |  |  |  |
| 2 | Existence of ongoing research activities |  |  |  |  |  |  |
| 3 | Existence of recent publications or about to be submitted manuscripts in peer reviewed journals |  |  |  |  |  |  |
| 4 | Existence of regular forums/meetings for sharing research activities |  |  |  |  |  |  |
| 5 | Existence of scheduled research capacity building |  |  |  |  |  |  |
| 6 | Existence of research proposal under development and/or submitted |  |  |  |  |  |  |

**Section 3. Research involvement/engagement**

1. How is your organization involved in research (Multiple responses)
   1. In what types of research is your organization/Team currently involved?
      1. Health System and Policy-related research
      2. Health services research other than clinical trials (e.g., monitoring and evaluation of health services).
      3. Behavioral or sociological research (e.g., substance use among urban youth).
      4. Clinical trials (e.g., testing patient safety and efficacy of a new drug).
      5. Epidemiological research (e.g., Outbreak investigations)
   2. To what extent is your organization/Team currently involved in research (Multiple responses)?
      1. We conduct research in partnership with one or more institutions (e.g., with a faculty member at a university)
      2. We conduct research in partnership with CSOs or NGOs
      3. We conduct research on our own
      4. We advise on research being conducted by others (e.g., serving on a study advisory committee)
      5. We endorse or support research being conducted by others (e.g., writing a letter of support for a study, allowing research to be conducted at our organization)
      6. We participate in research training being conducted by others (e.g., serving as a site for a multisite study)
   3. What partnerships/collaborations in research does your organization have?
      1. What type of collaborators/partners is your organization working with?
         1. Local partners
         2. International partners
         3. Both Local and International partners
         4. None
      2. What is the nature of the partnership/collaboration?
         1. Technical collaboration
         2. Financial support
         3. Both technical and financial
         4. None

**Section 4. Importance, motivations and priority for research**

1. How Important is research in your organization/team?
2. Not very important
3. Important
4. Moderately important
5. Somewhat important
6. Not very important
7. What are the main motivations for your organization wanting to develop its ability to conduct research or partner with academic institutions (Multiple responses)?
8. To use data for policy
9. To use data for local improvement of services
10. To bring in additional resources
11. To develop programs
12. To evaluate the effectiveness of programs
13. To monitor productivity
14. To support staff
15. In a scale of 0 to 5 indicate whether the following items are priority areas for research training in your organization

|  | **Priority areas for research** | **0** | **1** | **2** | **3** | **4** | **5** |
| --- | --- | --- | --- | --- | --- | --- | --- |
| 1 | Research methodology |  |  |  |  |  |  |
| 2 | Software (e.g. STATA, SAS, Nvivo, etc) |  |  |  |  |  |  |
| 3 | Existence of regular forums/meetings for sharing research activities |  |  |  |  |  |  |
| 4 | Setting research priorities using participatory approaches |  |  |  |  |  |  |
| 5 | Developing a research proposal/scientific writing |  |  |  |  |  |  |
| 6 | Data collection |  |  |  |  |  |  |
| 7 | Data analysis |  |  |  |  |  |  |
| 8 | Evaluating or reviewing research results |  |  |  |  |  |  |
| 9 | Developing a research proposal for funding |  |  |  |  |  |  |
| 10 | Evaluating research proposals |  |  |  |  |  |  |
| 11 | Preparing and submitting scientific papers for publication |  |  |  |  |  |  |
| 12 | Managing health research projects |  |  |  |  |  |  |
| 13 | Teaching or training others to produce research (facilitation and mentorship skills) |  |  |  |  |  |  |
| 14 | Training others to utilize research results (for decision making) |  |  |  |  |  |  |
| 15 | Reviewing or monitoring ethical aspects of research (Ethics in research) |  |  |  |  |  |  |
| 16 | Preparing policy brief for policy and decision making |  |  |  |  |  |  |
| 17 | Developing new interventions, practices from evidence |  |  |  |  |  |  |
| 18 | Preparing media briefs to the general public |  |  |  |  |  |  |
| 19 | Obtaining patents or product development |  |  |  |  |  |  |

**Section 5. Elements of effective trainings**

1. What are important elements/aspects to be considered for the proposed research training program to be effective in your organization***?***
2. **Participants selection**. Which level of staff member would you be willing to send to a research training program?
3. Hospital director/In-charges/ manager
4. Associate/coordinator of various programs, projects or interventions
5. Head of Departments/Sections or Units
6. Specialist Doctors
7. Doctors (general practitioners (MD/DDS)
8. Nursing Officer
9. Assistant Nursing Officers
10. Laboratory staff
11. Pharmacists
12. Other Healthcare providers
13. Not willing to send any staff member
14. **Time allocated for training.** How much of your time, or a staff member’s time, would you be willing to dedicate to a research training program?
15. One full day per week, over several months
16. Two or 3 full-day intensive
17. One-week intensive
18. **Training methodology**. In a scale of 0-5 how effective do you think the following methods of training would be in achieving the learning goals?

|  | **Effectiveness of training methodology** | **0** | **1** | **2** | **3** | **4** | **5** |
| --- | --- | --- | --- | --- | --- | --- | --- |
| 1 | Workshops/classroom setting |  |  |  |  |  |  |
| 2 | Webinar (e.g., Zoom |  |  |  |  |  |  |
| 3 | Toolkit/resource materials |  |  |  |  |  |  |
| 4 | Mentorship for research |  |  |  |  |  |  |
| 5 | On the job training |  |  |  |  |  |  |

1. What do you consider as critical factors for success or failure in conducting research?
2. In a scale of 0 to 5 what are the biggest barriers to research in your organization?

|  | **Barriers for conducting research** | **0** | **1** | **2** | **3** | **4** | **5** |
| --- | --- | --- | --- | --- | --- | --- | --- |
| 1 | Inadequate technical expertise-Research proposal writing |  |  |  |  |  |  |
| 2 | Inadequate technical expertise-Data analysis |  |  |  |  |  |  |
| 3 | Inadequate technical expertise-Scientific writings |  |  |  |  |  |  |
| 4 | Inadequate motivation |  |  |  |  |  |  |
| 5 | Inadequate financial resources |  |  |  |  |  |  |
| 6 | Inadequate infrastructure to support research |  |  |  |  |  |  |
| 7 | Inadequate data |  |  |  |  |  |  |
| 8 | Inadequate time |  |  |  |  |  |  |
| 9 | Others (specify) |  |  |  |  |  |  |

1. In a scale of 0 to 5 what are the most important facilitators to research?

|  | **Facilitators for conducting research** | **0** | **1** | **2** | **3** | **4** | **5** |
| --- | --- | --- | --- | --- | --- | --- | --- |
| 1 | Technical expertise-Research proposal writing |  |  |  |  |  |  |
| 2 | Technical expertise-Data analysis |  |  |  |  |  |  |
| 3 | Technical expertise-Scientific writings |  |  |  |  |  |  |
| 4 | Motivation |  |  |  |  |  |  |
| 5 | Financial resources |  |  |  |  |  |  |
| 6 | Infrastructure to support research |  |  |  |  |  |  |
| 7 | Rich availability of data |  |  |  |  |  |  |
| 8 | Time |  |  |  |  |  |  |
| 9 | Other (specify) |  |  |  |  |  |  |
