# Supplementary material for: Research capacity, motivators and barriers to conducting research among healthcare providers in Tanzania’s public health system: a mixed methods study
Source: Hum Resour Health. 2023 Sep 5;21:73. doi: 10.1186/s12960-023-00858-w (PMC10478476; doi:10.1186/s12960-023-00858-w)
Supplement: Supplementary file 4 — Additional file 4. Interview protocol. [file 12960_2023_858_MOESM4_ESM.doc]

**Additional file 4**. Interview protocol

1. Please describe your role/function in the facility/team or organization.
2. Could you describe how your facility/team or organization participate sin research.

Probes

- 1. Is your facility/team or organization engaged in research?
  2. What types of research the facility/team or organization is engaged?
  3. To what extent is your facility engaged in research?

1. What are your main motivations for conducting research?
2. What research structures/supports/processes currently exist within your organization, facility or team?

Probes

- 1. Is there specific unit/section or department coordinating research?
  2. Is there specific person assigned research coordination roles?
  3. Is there specific budget allocated for research activities?
  4. Is there guidelines or protocols to support research activities?
  5. Do you have a Memorandum of understanding from nearby University
  6. Do you have support from National Institute for Medical Research (NIMR)?

1. What are the biggest barriers for conducting research in your organization/facility/team?

Probes

- 1. Lack of knowledge or skills
  2. Lack of time
  3. Lack of resources
  4. Lack of motivation
  5. Not part of job description

1. What opportunities for capacity building in research are available in your organization?

Probes

- 1. The presence of any organization support research activities
  2. The presence of trained individuals in research
  3. The presence of teaching materials for research

1. What are your main priorities for training in research? What competencies are most important to develop?
2. How much time would you be willing to dedicate to a research training program?
3. What is the most effective way of delivering research capacity building program?
4. What do you consider to be the critical factors in establishing a strong research capacity building program?
5. How can the research culture in this facility/team or organization be strengthened?
6. We want to understand how to implement change successfully in your organization
   - How easy is it to initiate change in your organization?
   - Who are the innovators within your organization?
   - How is best practice shared across the organization?
   - How are the staff developed within the organization?
   - How does the organization reward innovation?
7. Is there anything that has not be covered in this discussion and you want to explain?

**Specific questions for RHMTs and ministries officials**

1. What is your opinion with regards to design and implementation of research capacity building program for lower levels that you are supervising?

Probe

1. Priority areas for research capacity building
2. Modality of conducting training (Time, face to face or Zoom e.t.c)
3. Participants for the trainings
4. What support could you provide to ensure success in health research capacity building program for the lower levels institutions, facilities or teams that you are supervising?
