# Supplementary material for: Research capacity, motivators and barriers to conducting research among healthcare providers in Tanzania’s public health system: a mixed methods study
Source: Hum Resour Health. 2023 Sep 5;21:73. doi: 10.1186/s12960-023-00858-w (PMC10478476; doi:10.1186/s12960-023-00858-w)
Supplement: Supplementary file 5 — Additional file 5. Experience and confidence in conducting research activities. [file 12960_2023_858_MOESM5_ESM.doc]

**Additional file 5.** Experience and confidence in conducting research activities

| **Experience and confidence of healthcare workers (N=209)** | **Experience**  **n(%)** | **Confidence**  **n(%)** |
| --- | --- | --- |
| Finding relevant literature | 128(61.2%) | 106(50.7%) |
| Critically reviewing literature | 102(48.8%) | 74(35.4%) |
| Generating research ideas | 108(51.7%) | 81(38.8%) |
| Writing a research proposal | 110(52.6%) | 92(44%) |
| Research ethics | 71(34%) | 46(22%) |
| Using research software | 74(35.4%) | 53(25.4%) |
| Using quantitative research methods | 71(34%) | 60(28.7%) |
| Using qualitative research methods | 72(34.5%) | 41(19.6%) |
| Using mixed methods research design | 53(25.4%) | 35(16.8%) |
| Applying for research funding | 25(12%) | 16(7.7%) |
| Analyzing and interpreting results | 59(28.2%) | 47(22.5%) |
| Writing and presenting abstract or paper | 45(21.5%) | 35(16.8%) |
| Writing and publishing research | 39(18.7%) | 21(10.1%) |
| Managing a research project | 26(12.4%) | 19(9.1%) |
