# Supplementary material for: Research capacity, motivators and barriers to conducting research among healthcare providers in Tanzania’s public health system: a mixed methods study
Source: Hum Resour Health. 2023 Sep 5;21:73. doi: 10.1186/s12960-023-00858-w (PMC10478476; doi:10.1186/s12960-023-00858-w)
Supplement: Supplementary file 6 — Additional file 6. Priorities for developing future research capacity. [file 12960_2023_858_MOESM6_ESM.doc]

**Additional file 6.** Areas of interest in research capacity building

| **Areas of interest in research capacity building (N=443)** | **None/ Very low**  **n(%)** | **Low/Medium**  **n(%)** | **High/Very High**  **n(%)** |
| --- | --- | --- | --- |
| Finding relevant literature | 19(4.3%) | 142(32.1%) | 282(63.7%) |
| Critically reviewing literature | 16(3.6%) | 131(29.6%) | 296(66.8%) |
| Generating research ideas | 10(2.3%) | 112(25.3%) | 321 (72.5%) |
| Writing a research proposal | 7(1.6%) | 105(23.7%) | 331 (74.7%) |
| Research ethics | 14(3.2%) | 124(28%) | 305 (68.8%) |
| Using research software | 9(2%) | 142(32.1%) | 292 (65.9%) |
| Using quantitative research methods | 7(1.6%) | 109(24.6%) | 327(73.8%) |
| Using qualitative research methods | 10(2.3%) | 122(27.5%) | 311(70.2%) |
| Using mixed methods research design | 11(2.5%) | 108(24.4%) | 324(73.1%) |
| Applying for research funding | 7(1.6%) | 73(16.5%) | 363(81.9%) |
| Analyzing and interpreting results | 4(0.9%) | 111(25.1%) | 328(74%) |
| Writing and presenting abstract | 8(1.8%) | 101(22.8%) | 334(75.4%) |
| Writing and publishing research | 4(0.9%) | 81(18.3%) | 358(80.8%) |
| Managing a research project | 4(0.9%) | 84(19%) | 355(80.1%) |
