# Supplementary material for: Research capacity, motivators and barriers to conducting research among healthcare providers in Tanzania’s public health system: a mixed methods study
Source: Hum Resour Health. 2023 Sep 5;21:73. doi: 10.1186/s12960-023-00858-w (PMC10478476; doi:10.1186/s12960-023-00858-w)
Supplement: Supplementary file 7 — Additional file 7. Percentage of respondents who reported receiving research training by education level and age group. [file 12960_2023_858_MOESM7_ESM.doc]

**Additional file 7.** Percentage of respondents who reported receiving research training by education level and age group

| **Education level** | **Underwent research training** | **Age Group** | | | |
| --- | --- | --- | --- | --- | --- |
|  |  | 23-30 years  **n(%)** | 31-35 years  **n(%)** | 36-40 years  **n(%)** | 40+ years  **n(%)** |
| Certificate |  |  |  |  |  |
|  | Yes | 1(2.86%) | 2(11.76%) | 0(0.00%) | 2(14.29%) |
|  | No | 34(97.14%) | 15(88.24%) | 6(100.00%) | 12(85.71%) |
| Undergraduate degree |  |  |  |  |  |
|  | Yes | 36(59.02%) | 22(36.07%) | 14(31.11%) | 26(57.78%) |
|  | No | 25(40.98%) | 39(63.93%) | 31(68.89%) | 19(42.22%) |
| Postgraduate degree |  |  |  |  |  |
|  | Yes | 10(43.48%) | 20(48.78%) | 26(56.52%) | 45(66.18%) |
|  | No | 13(56.52%) | 21(51.22%) | 20(43.48%) | 23(33.82%) |
